# EA participates in pain transition through regulating KCC2 expression by BDNF-TrkB in the spinal cord dorsal horn of male rats

**DOI:** 10.1016/j.ynpai.2023.100115

**Published:** 2023-01-05

**Authors:** Mengting Shi, Jie Zhou, Rong Hu, Haipeng Xu, Yi Chen, Xingying Wu, Bowen Chen, Ruijie Ma

**Affiliations:** a The Third School of Clinical Medicine, Zhejiang Chinese Medical University, Key Laboratory of Acupuncture and Neurology of Zhejiang Province, Hangzhou, Zhejiang, China; b Department of Acupuncture and Moxibustion, Third Affiliated Hospital of Zhejiang Chinese Medical University, Hangzhou, Zhejiang, China

**Keywords:** Pain transition, Electroacupuncture, Hyperalgesic priming, Brain derived neurotrophic factor

## Abstract

The pathogenesis of chronic pain is complex and poorly treated, seriously affecting the quality of life of patients. Electroacupuncture (EA) relieves pain by preventing the transition of acute pain into chronic pain, but its mechanism of action is still unclear. Here, we aimed to investigate whether EA can inhibit pain transition by increasing KCC2 expression via BDNF-TrkB. We used hyperalgesic priming (HP) model to investigate the potential central mechanisms of EA intervention on pain transition. HP model male rats showed significant and persistent mechanically abnormal pain. Brain derived neurotrophic factor (BDNF) expression and Tropomyosin receptor kinase B (TrkB) phosphorylation were upregulated in the affected spinal cord dorsal horn (SCDH) of HP model rats, accompanied by K^+^-Cl^-^- Cotransporter-2 (KCC2) expression was down-regulated. EA significantly increased the mechanical pain threshold in HP model male rats and decreased BDNF and p-TrkB overexpression and upregulated KCC2 expression. Blockade of BDNF with BDNF neutralizing antibody attenuated mechanical abnormal pain in HP rats. Finally, administration of exogenous BDNF by pharmacological methods reversed the EA-induced resistance to abnormal pain. In all, these results suggest that BDNF-TrkB contributes to mechanical abnormal pain in HP model rats and that EA ameliorates mechanical abnormal pain through upregulation of KCC2 by BDNF-TrkB in SCDH. Our study further supports EA as an effective treatment to prevent the transition of acute pain into chronic pain.

## Highlights

• This study observed the mechanism of electroacupuncture (EA) in preventing acute pain from turning into chronic pain.
• The model of hyperalgesic priming (HP) was used to study whether electroacupuncture could pass BDNF TrkB by activating KCC2.
• Through pharmacological methods, exogenous BDNF was given to reverse the therapeutic effect of EA on abnormal pain.

## 1 Introduction

For a long time, chronic pain has seriously affected patients' physical and mental health and quality of life, and is a major problem in clinical treatment. The pathogenesis of chronic pain is complex, and the efficacy of existing pharmacological treatments is not ideal (Kaye et al., 2017). In the transition of acute pain to chronic pain, the pathogenesis of pain will change significantly (Reichling and Levine, 2009), so in addition to directly targeting chronic pain, preventing the transition of acute pain to chronic pain is another idea for chronic pain treatment (Jungquist et al., 2017). In conclusion, it is of great interest to deeply uncover the mechanisms of pain transition and to seek effective treatments.

Electroacupuncture (EA) is an effective analgesic method that has been widely used for clinical pain control and scientific research on this condition (Heo et al., 2021). The current study (Wang et al., 2021) showed that EA treatment blocked the production of nociceptive sensitization evoked and contributed to the normalization of mechanical withdrawal threshold (MWT) in nociceptive sensitized rats. Some studies have also demonstrated that EA at the Zusanli (ST36) and Kunlun (BL60) acupoints can intervene in pain transition (Wang et al., 2020). Modern studies have shown that the Zusanli (ST36) and Kunlun (BL60) acupoints are an effective combination of acupuncture points for EA in the treatment of chronic inflammatory pain (Du et al., 2017). Although the effect of EA on blocking nociceptive sensitization has been well demonstrated, the analgesic mechanism of action remains largely unexplored.

Brain derived neurotrophic factor (BDNF) plays an important role in neuronal survival, differentiation, and growth and development (Song et al., 2017, Khalin et al., 2015). Tropomyosin receptor kinase B (TrkB) is a strong affinity receptor for BDNF (Allen et al., 2013). K^+^-Cl^-^-Cotransporter-2 (KCC2) is the major chloride transporter protein in the spinal dorsal horn and determines the differential transmembrane electrochemical gradient of chloride ions in neurons (Liu et al., 2019). In existing studies, the BDNF-TrkB signaling pathway, plays an important role in neurological disorders such as epilepsy, Parkinson's, and Alzheimer's syndrome (Harward et al., 2016, Lanuza et al., 2019, Lin et al., 2018). After nerve injury, the BDNF-TrkB signaling pathway downregulates KCC2 expression in the dorsal horn of the spinal cord, and the inhibitory synaptic effects are diminished to induce pain (Wang et al., 2009, Miletic and Miletic, 2008). It has also been shown that morphine-induced nociceptive hyperalgesia is induced by the release of BDNF in microglia, which downregulates KCC2 expression in SCDH through TrkB receptor action (Ferrini et al., 2013). EA suppresses chronic pain through upregulation of KCC2 expression in the dorsal horn of the spinal cord (Li et al., 2018). Therefore, we hypothesized that EA may exert analgesic effects by interfering with the expression of KCC2 in the spinal cord through BDNF-TrkB to prevent pain transition.

In this study, we used David B. Reichling and Jon D. Levine's hyperalgesic priming (HP) rats model of the transition from acute to chronic pain originally developed (Reichling and Levine, 2009). We investigated whether KCC2 was inhibited in the spinal cord of pain-transformed rats and the role of BDNF in regulating KCC2. We further explored whether EA intervention affects KCC2 expression and examined whether EA activates KCC2 in the spinal cord via BDNF by pharmacological methods. This study provides new evidence that EA can be involved in pain transition through regulating KCC2 by BDNF-TrkB in the dorsal horn of spinal cord.

## 2 Materials and methods

### 2.1 Animals

All experimental procedures were approved by the Animal Care and Welfare Committee of Zhejiang University of Traditional Chinese Medicine, Zhejiang, China (approval number IACUC-20180319–12). Male Sprague-Dawley rats (weighing 200–230 g) used in this study were obtained from the Shanghai Experimental Animal Center of the Chinese Academy of Sciences [SCXK(Hu)2018–0006] and housed at the Experimental Animal Center of Zhejiang University of Traditional Chinese Medicine [SYXK(Zhejiang)2018–0012]. All rats were fed standard rodent food, housed five per cage, and regularly cycled in light and dark. Previous studies have shown that there was a genderdependent difference in hyperalgesic priming, and PGE2 induced hyperalgesia was prolonged to 24 h in male rates treated 5 days prior with Car that did not induce hyperalgesia in female rats (Joseph et al., 2003). Therefore, only male rats were selected for the experiment in this study.

### 2.2 Drug preparation

Carrageenan (Car) and prostaglandin E 2 (PGE 2) were purchased from Sigma-Aldrich (St. Louis, MO, United States). BDNF neutralizing antibody (#AB1513P) was purchased from Millipore, and BDNF recombinant protein (AF-450–02) was purchased from PeproTech.

A stock solution of PGE2 (1 μg/μL) was prepared in 10 % ethanol and dissolved in saline (NS) to a concentration of 100 ng/25 μL immediately prior to injection. A stock solution of Car (2 %) was dissolved in NS. A stock solution of BDNF neutralizing antibody (0.5 μg/μL) was prepared in PBS and dissolved to a concentration of 0.05 μg/μL immediately prior to injection. A stock solution of BDNF recombinant protein (1 μg/μL) was prepared in ultrapure water and dissolved to a concentration of 0.02 μg/μL immediately prior to injection.

### 2.3 Hyperalgesic priming model

HP model was established by injecting 100 μL of 1 % carrageenan into the left plantar (first injection) and 25 μL of PGE 2 into the left dorsum of the foot (second injection) at seven days after the first injection. In the sham HP group of rats, the same volume of NS was given instead of Car.

### 2.4 Mechanical withdrawal threshold

The up-down method was used in this study (Chaplan et al., 1994). The force used was 0. 4, 0. 6, 1, 2, 4, 6, 8, 15 and 26 g of Von Frey filament (Stoelting Co, Thermo, Gilroy, CA, United States). The rats were placed in clear plastic cages for 30 min each day before assessment for three days to acclimatize. A von Frey filament of 4 g force was first applied to the central surface of the hind paw (avoiding the foot pads) until the filament bent into an “S” shape and was held there for 6 s. The filament was then selected for greater or lesser force depending on whether the response was negative or positive. The response is recorded as X or O. The pain threshold is calculated according to the following equation: Mechanical retreat threshold (MWT) (g) = [10 (Xf + κδ)]/10,000, where “Xf” is the force of the last hair test, “κ” is the value obtained from the k-value table, and “δ” is the average of the difference of the logarithm of hairs for each force, which is approximately equal to 0. 231.

### 2.5 EA intervention

Following the first injection, EA intervention was initiated 4 h after the first injection, and was repeated once a day until the end of the experiment (Wang et al., 2021). The 0.18 mm × 13 mm acupuncture needles were inserted into the bilateral foot Zusanli (ST36) and Kunlun (BL60) at a depth of 5 mm, and then connected to a HANS acupoint nerve stimulator (HANS-200A Huawei Co., Beijing, China). The stimulation parameters were as follows: 2/100 Hz and 0.5 mA, 1.0 mA and 1.5 mA intensity (intensity increased every 10 min) for a total of 30 min (Fang et al., 2021).

### 2.6 Sham EA intervention

Sham EA was also performed. Needles of the same size were inserted subcutaneously into the animals at the ST36 and BL60 points (depth of 1 mm). The needles were connected to the same stimulator, but no electrical stimulation was performed.

### 2.7 Intrathecal drug delivery

The drug was applied through a lumbar catheter insertion. Rats were first anesthetized with sodium pentobarbital (50 mg/kg, ip). The lumbar region was shaved and disinfected with 75 % ethanol. A small incision was made along the L4-5 lumbar spine. The intervertebral ligaments were cut to expose the intervertebral foramen. A PE-10 catheter pre-filled with sterile PBS is inserted into the subarachnoid space. Proper insertion of the catheter is demonstrated by tailing or claw retraction. After insertion, the position of the catheter in the spinal canal was checked by lidocaine (1 %, 10 μL/rat) injection. Correct insertion of the catheter is demonstrated by rapid motor paralysis of the hind limbs, usually lasting 15–30 min. The catheter is secured and the skin incision is sutured. The rats are then placed back in a separate cage for recovery and receive close monitoring. Intrathecal dosing is administered for 3 consecutive days starting on the day of PGE2 injection, with each injection consisting of 0.05 μg/μL of BDNF neutralizing antibody in 10 μL PBS (total 0.5 μg). Each injection consists of 0.02 μg/μL of BDNF recombinant protein in 10 μL of matching alginate solution (total 0. 2 μg). IgG and PBS equal to the intervention drug were given as controls for BDNF-neutralizing antibody and BDNF recombinant protein, respectively.

### 2.8 Immunofluorescence

Rats were executed 48 h after the 2nd injection after completion of MWT assessment. Rats were anesthetized with sodium pentobarbital (50 mg/kg, ip). Then, the rats were treated with 0. The rats were rapidly perfused with 9 % NaCl (4 °C), followed by 0. 1 M phosphate-buffered saline (PBS) in 4 % paraformaldehyde for preperfusion. L4-L6 spinal cords were removed and post-fixed in 4 % paraformaldehyde at 4 °C for 3 h, then transferred to 15 % and 30 % sucrose for dehydration and stored in a −80 °C refrigerator. The spinal cord was sectioned transversely (10 μm) using a cryostat and dried at 37 °C for 20 min. Sections were then blocked with 10 % goat serum in TBST (1 % Tween-20) at 37 °C for 1 h. The sections are then incubated overnight at 4 °C with primary antibodies diluted in the blocking solution. Primary antibodies were mouse anti-BDNF (66292–1-Ig, Proteintech), rabbit anti-p-TrkB (NBP1-03499, NOVUS), and rabbit anti-KCC2 (94725, Cell Signaling Technology). Sections were then washed with TBST and incubated with goat anti-rabbit (Alexa Fluor 488 marker, 111–545-144, Jackson Immunoresearch) or goat anti-mouse (Alexa Fluor 594 marker, ab150120, abcam) secondary antibodies for 1 h at 37 °C (with 10 % goat serum (diluted in TBST). Images were taken using an M2 microscope (Zeiss, Germany) Imager. Analysts were blinded to the experimental design.

### 2.9 Western blotting

To measure protein expression of BDNF, TrkB and KCC2, rats were deeply anesthetized after the last behavioral test, as described previously. The L4-L6 affected dorsal horn of the spinal cord was quickly excised and stored at −80 °C for further analysis. Samples were then lysed with RIPA lysis buffer to lyse the tissue and centrifuged at 12,000 rpm for 12 min at 4 °C. The supernatant was collected and protein concentration was measured using the BCA Protein Assay Kit. Equal amounts of protein samples (20μg) were separated on 5 % SDS-PAGE gels and transferred to PVDF membranes. After being closed with 5 % skim milk for 1 h at room temperature, the membranes were incubated with mouse anti-BDNF (1:1000, 66292–1-Ig, Proteintech), rabbit anti-KCC2 (1:1000, 94725, Cell Signaling Technology) as primary antibody and mouse anti-β-actin (HRP coupled) (1. 5000, ab20272, Abcam) as an internal control, while rabbit anti-p-TrkB (1:1000, NBP1-03499, NOVUS) was used as primary antibody and rabbit anti-TrkB (1:1000, 4603S, Cell Signaling Technology) as control at 4 °C overnight. The membranes were then incubated with horseradish peroxidase (HRP) conjugated goat anti-rabbit IgG (1:5000, 7074S, Abcam) or goat anti-mouse (1:5000, BK-M050, Bioker biotechnology) for 1 h at room temperature. Signals were developed using an ECL kit (Pierce, Rockford, IL, USA) and analyzed using ImageQuant TL 7. 0 analysis software (GE, USA) to analyze the intensity of the bands. Analysts were blinded to the experimental design.

### 2.10 qPCR

Rats were deeply anesthetized using pentobarbital (50 mg/kg, i.p.) and transcardially perfused with 200 mL normal saline (4 °C). The spinal cord was immediately removed and stored at − 80 °C. The expression of the 10 hub genes was verified by qRT-PCR. Total RNA was extracted from the spinal cord tissue using Trizol reagent (Invitrogen, Carlsbad, USA) according to the manufacturer’s protocol. Primer sequences are listed in Table 1. qPCR was performed using the Fast Start Universal SYBR Green Master kit (TaKaRa Bio Inc, China) with a 25 μL reaction system according to the manufacturer’s protocol by CFX96 Real-Time System (BioRad, USA). Each reaction was performed in triplicate and normalized to gapdh gene expression. The CT value of each well was determined using the CFX96 Real-Time System software and the average of the triplicates was calculated. The relative quantification was determined by ΔΔCT method. The primer sequences were shown in Table 1.

**Table 1:** Sequences of the primers used for qPCR.

Gene name	Primer Sequence (5′-3′)	Amplicon size (bp)
BDNF	F: TGGAACTCGCAATGCCGAACTAC	88
	R: TCCTTATGAACCGCCAGCCAATTC	
Ntrk2	F:GGTCTATGCCGTGGTGGTGATTG	82
(TrkB)	R: ATGTCTCGCCAACTTGAGCAGAAG	
Slc12a5	F: AGGGAAGCAAAGAGCACGAAGAAG	144
(KCC2)	R: GAGCCGCAGAAAGAGGGATAACAC	

### 2.11 Experimental design and groups

**Fig. 1:**
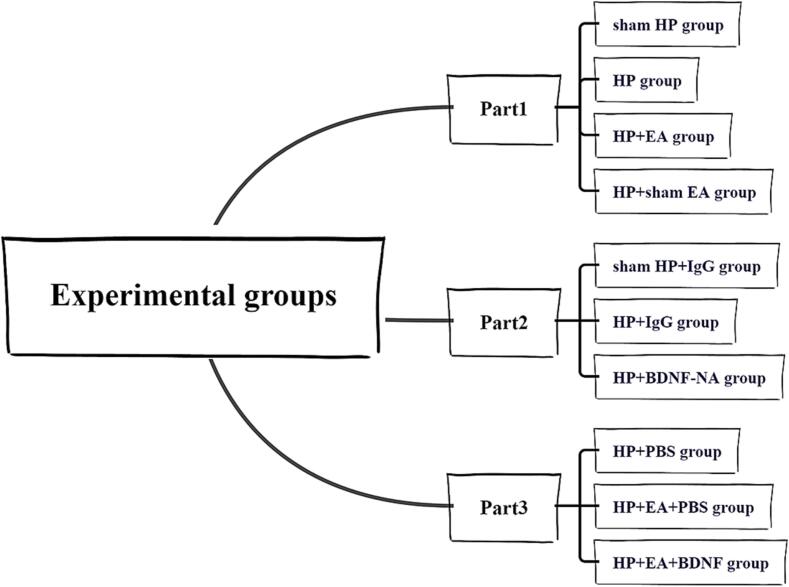
The Experimental groups of the whole study divided into three parts.

In this study, we sought to demonstrate the effect of EA on pain transition in three parts (Fig. 1). First, we attempted to identify the central mechanisms of pain shifts using a nociceptive hyperalgesia initiation model. We observed the changes in MWTs in rats to ensure that we had successfully established the model. In this part, rats were randomly divided into four groups: sham HP group (NS + PGE2), HP group (Car + PGE2), HP + EA group (EA intervention given during model establishment) and HP + sham EA group (sham EA intervention given during model establishment). The expression levels of BDNF, TrkB and KCC2 were also investigated to identify possible EA intervention pathways. We then selectively inhibited the expression of BDNF, suggesting that they are all involved in PGE2-induced nociceptive hyperalgesia. In this section, rats were divided into three groups: sham HP + IgG group (NS + PGE2, intrathecal administration of IgG), HP + IgG group (Car + PGE2, intrathecal administration of IgG), and HP + BDNF-NA group (Car + PGE2, intrathecal administration of BDNF neutralizing antibody an hour before PGE2 injection, 23 h and 47 h after PGE2 injection). Finally, we gave BDNF recombinant protein for 3 consecutive days after 7 days of EA intervention to demonstrate that EA plays an important role in the transition from acute to chronic pain through regulating KCC2 by BDNF-TrkB in the dorsal horn of spinal cord. In this part, rats were randomly divided into three groups, HP + PBS group (Car + PGE2, intrathecal administration of PBS), HP + EA + PBS group (Car + PGE2, EA intervention and intrathecal administration of PBS), and HP + EA + BDNF group (Car + PGE2, EA intervention and intrathecal administration of BDNF recombinant protein 3 h, 23 h and 47 h after PGE2 injection). We also investigated the changes in MWT and the differences in BDNF, TrkB and KCC2 expression between this part of the groups.

### 2.12 Statistical analysis

The statistical analyses between control and experimental groups were completed by GraphPad Prism7 and were analyzed with a One-way ANOVA followed by Tukey Kramer test. Results are expressed as mean ± SEM. The results were considered to indicate statistically significant when *p* < 0.05.

## 3 Results

### 3.1 Establishment of HP model and EA intervention reduced mechanical pain

**Fig. 2:**
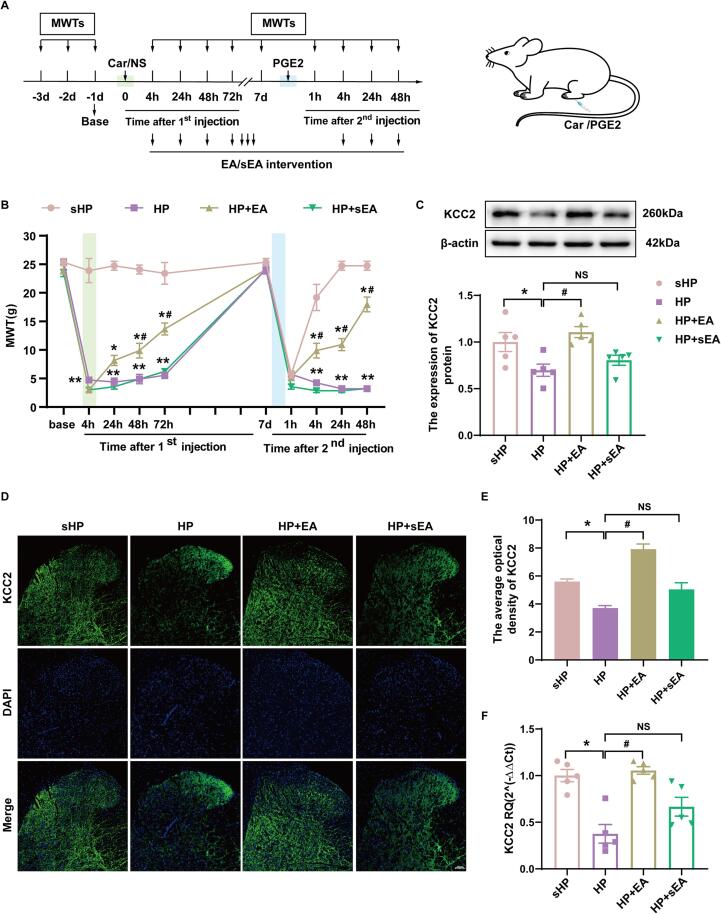
KCC2 in the L4-L6 SCDH plays a key role in the hyperalgesic priming model. (A) Schedule of establishing hyperalgesic priming model and EA/sham EA intervention. The hyperalgesic priming model was established by sequential intraplantar injection of carrageenan (Green arrow, 1 mg/100 μL/paw) and PGE2 (Blue arrow, 100 ng/25 μL/paw) into the left hind paw. (B) The mechanical withdrawal threshold (MWT) of rats that received carrageenan (Green line) and PGE2 (Blue line) injections. (C) The quantification of the Western blots results and a representative Western blot showing KCC2 protein isolated from the ipsilateral SCDH 48 h after 2nd injection. (D) Representative immunofluorescence images of KCC2 (green) merged with Dapi (blue) in ipsilateral L4-6 SCDH of hyperalgesic priming rats. (E) The immunofluorescence quantification of KCC2 in ipsilateral L4-6 SCDH. (F) Evaluation of KCC2 gene expression in ipsilateral L4-6 SCDH using qPCR in the sHP, HP, HP + EA, HP + sEA groups 48 h after 2nd injection. Data are presented as mean ± SEM, n = 5; *p < 0.05, **p < 0.01 vs sHP group, ^#^*p* < 0.05, ^##^*p* < 0.01 vs HP group. Two-way ANOVA with Tukey's post-hoc test was applied in. (For interpretation of the references to colour in this figure legend, the reader is referred to the web version of this article.)

We first established HP model according to the previously described method (Fig. 2A). By injecting 100 μL of 1 % Car into the plantar aspect of the left hind paw of rats and 25 μL of PGE2 into the dorsal aspect of the left hind paw 7 days later, we found that rats in the HP group displayed remarkable mechanical allodynia in the ipsilateral hind paws. This indication is consistent with previous studies and suggests that the hyperalgesic priming model was successfully established. We then investigated the therapeutic effect of EA on mechanical abnormal pain in the HP model rats. Fig. 1A illustrates the schedule of EA/sham EA intervention. In this study, we chose 2/100 Hz as the frequency of EA intervention. The EA intervention significantly contributed to the reduction of MWTs induced by carrageenan injection from 48 h to 72 h after the first injection (*p* < 0.05), and increased MWTs at 4 h, 24 h and 48 h after the second injection (*p* < 0.05). In contrast, there was no difference between the sham EA and HP groups, indicating that the EA intervention effectively reduced mechanical abnormal pain in the HP model rats (Fig. 2B, *p* < 0.05).

### 3.2 EA activates KCC2 in the affected dorsal horn of the spinal cord in hyperalgesic priming model rats

EA has been shown to inhibit chronic pain through upregulation of KCC2 expression (Yuan et al., 2022). Therefore, we aimed to investigate whether EA intervention affects the expression of KCC2 in the SCDH of hyperalgesic priming model rats. By Western blotting, we found that the protein expression of KCC2 was significantly decreased in the HP group compared to the sHP group in the ipsilateral SCDH. EA intervention significantly increased the protein expression of KCC2, while sham EA had no such effect (Fig. 2C, *p* < 0.05). In addition, immunofluorescence likewise showed (Fig. 2D-E, *p* < 0.05) that the mean fluorescence intensity of KCC2 was significantly decreased in the ipsilateral SCDH of rats in the HP group compared with the sHP group, and that EA intervention significantly activated the expression of KCC2 in HP model rats, whereas sham EA did not have this effect. Finally, qPCR results showed that gene expression of KCC2 was significantly decreased in the ipsilateral SCDH of the HP model rats. The gene expression of KCC2 was reversed in HP rats after EA intervention. These data suggest that EA effectively activated KCC2 in the SCDH of HP rats (Fig. 2F, *p* < 0.05).

### 3.3 EA inhibits BDNF expression and TrkB phosphorylation in the ipsilateral SCDH of the spinal cord in HP model rats

**Fig. 3:**
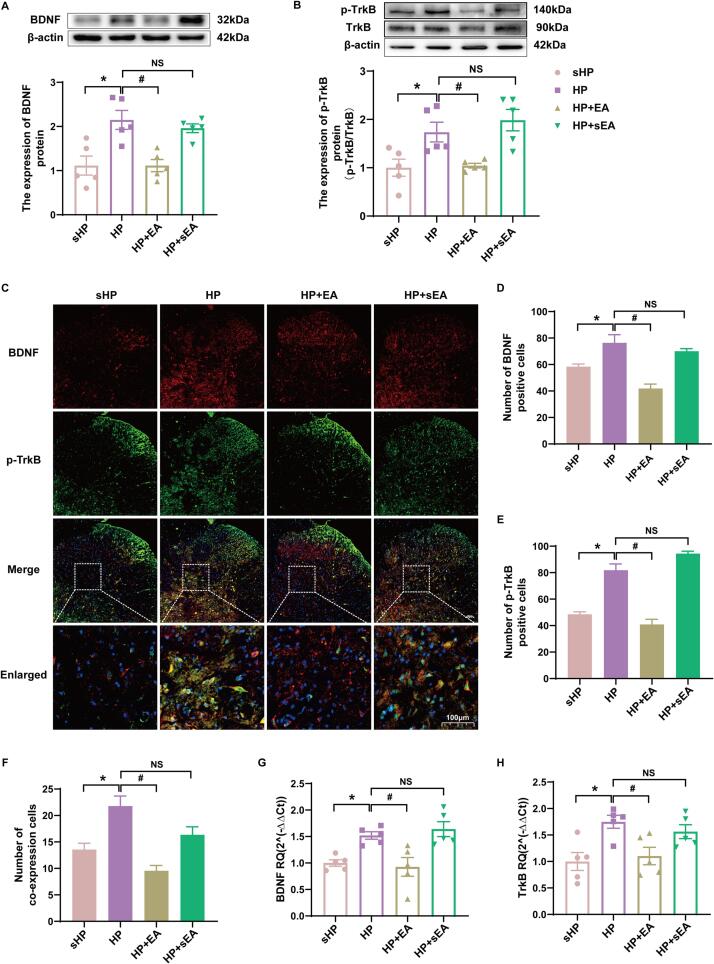
BDNF and TrkB in the L4-L6 SCDH plays a key role in the hyperalgesic priming model. (A-B) The quantification of the Western blots results and a representative Western blot showing BDNF and p-TrkB protein isolated from the ipsilateral SCDH 48 h after 2nd injection. (C) Representative immunofluorescence images of BDNF (red) merged with p-TrkB (green) in ipsilateral L4-6 SCDH of hyperalgesic priming rats. (D-F) The immunofluorescence quantification of BDNF and p-TrkB co-expression cells in ipsilateral L4-6 SCDH. (G-H) Evaluation of BDNF and p-TrkB gene expression in ipsilateral L4-6 SCDH using qPCR in the sHP, HP, HP + EA, HP + sEA groups 48 h after 2nd injection. Data are presented as mean ± SEM, n = 5; *p < 0.05, **p < 0.01 vs sHP group, ^#^*p* < 0.05, ^##^*p* < 0.01 vs HP group. Two-way ANOVA with Tukey's post-hoc test was applied in. (For interpretation of the references to colour in this figure legend, the reader is referred to the web version of this article.)

Blocking BDNF-TrkB signaling reduces neuropathic pain (Zhao et al., 2022). Therefore, we speculate that EA intervention may affect BDNF expression and TrkB phosphorylation in the SCDH of pain-transformed rats. Western blotting showed that protein expression of both BDNF and p-TrkB was significantly upregulated in the affected SCDH in the HP group, compared to the sHP group. EA intervention reduced the upregulation of BDNF and p-TrkB expression in the ipsilateral SCDH, whereas sham EA had no such effect (Fig. 3A-B, *p* < 0.05). In addition, immunofluorescence also showed a significant increase in the number of BDNF and p-TrkB co-expression cells in the ipsilateral SCDH of rats in the HP group compared with the sHP group, and EA intervention significantly inhibited the expression of BDNF and p-TrkB in HP model rats (Fig. 3C-F, *p* < 0.05). Finally, qPCR results showed that gene expression of BDNF and TrkB were significantly decreased in the ipsilateral SCDH of the HP model rats and gene expression of BDNF and TrkB was reversed after EA intervention (Fig. 3G-H, *p* < 0.05).

### 3.4 Neutralizing BDNF reduced mechanical pain in HP model rats and activates KCC2 in the SCDH

**Fig. 4:**
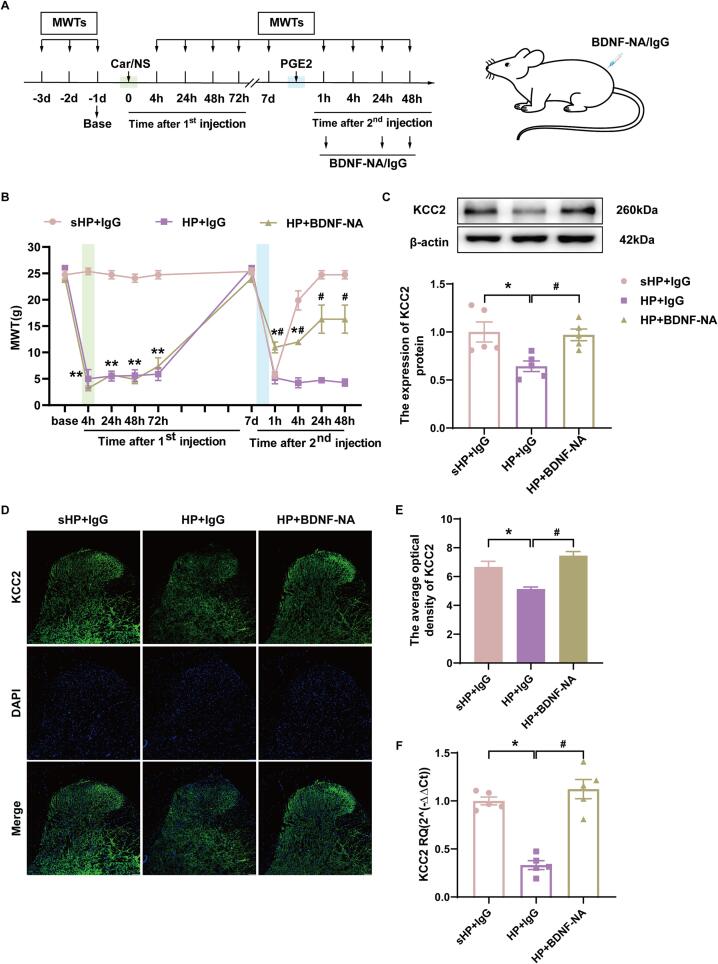
BDNF-neutralizing antibody intervention prevented the occurrence of hyperalgesic priming. (A) Schedule of establishing hyperalgesic priming model and BDNF-neutralizing antibody intervention. BDNF-neutralizing antibody or IgG was administered through the intrathecal catheter to HP model rats for 3 consecutive days starting on day 7, 1 h before the detection of mechanical withdrawal threshold. (B) The mechanical withdrawal threshold (MWT) of rats that received 3 consecutive days BDNF neutralizing antibody intervention. (C) The quantification of the Western blots results and a representative Western blot showing KCC2 protein isolated from the ipsilateral SCDH 48 h after 2nd injection. (D) Representative immunofluorescence images of KCC2 (green) merged with Dapi (blue) in ipsilateral L4-6 SCDH of BDNF neutralizing antibody intervention rats. (E) The immunofluorescence quantification of KCC2 in ipsilateral L4-6 SCDH. (F) Evaluation of KCC2 gene expression in ipsilateral L4-6 SCDH using qPCR in the sHP + IgG, HP + IgG, HP + BDNF neutralizing antibody groups 48 h after 2nd injection. Data are presented as mean ± SEM, n = 5; *p < 0.05, **p < 0.01 vs sHP + IgG group, ^#^*p* < 0.05, ^##^*p* < 0.01 vs HP + IgG group. Two-way ANOVA with Tukey's post-hoc test was applied in. (For interpretation of the references to colour in this figure legend, the reader is referred to the web version of this article.)

To determine whether BDNF activating KCC2 mediateed pain transition, we further intervened using BDNF neutralizing antibody. The role of BDNF in pain transition was examined by intrathecal infusion of BDNF neutralizing antibodies into the spinal cord using intrathecal cannulation. As shown in Fig. 4A, BDNF neutralizing antibody (0.5 μg/rat/day) or IgG was administered through the intrathecal catheter to HP model rats for 3 consecutive days starting on day 7, 1 h before the detection of mechanical pain threshold. The results showed that the BDNF neutralizing antibody partially reversed the mechanical pain threshold of sensitized rats (Fig. 4B, *p* < 0.05). Western blotting showed a significant increase in KCC2 protein expression in the HP + BDNF neutralizing antibody group compared to the HP group (Fig. 4C, *p* < 0.05). Immunofluorescence showed that the mean fluorescence intensity of KCC2 was significantly increased in the ipsilateral SCDH of rats in the HP + BDNF-neutralized antibody group compared with the HP + IgG group (Fig. 4D-E, *p* < 0.05). In addition, the qPCR results also showed that the gene expression of KCC2 in the ipsilateral SCDH of the spinal cord increased significantly after the BDNF neutralizing antibody intervention (Fig. 4F, *p* < 0.05). These results suggested that BDNF in the dorsal horn of the spinal cord of HP model rats regulates expression of KCC2.

### 3.5 Neutralizing BDNF reduced TrkB phosphorylation

**Fig. 5:**
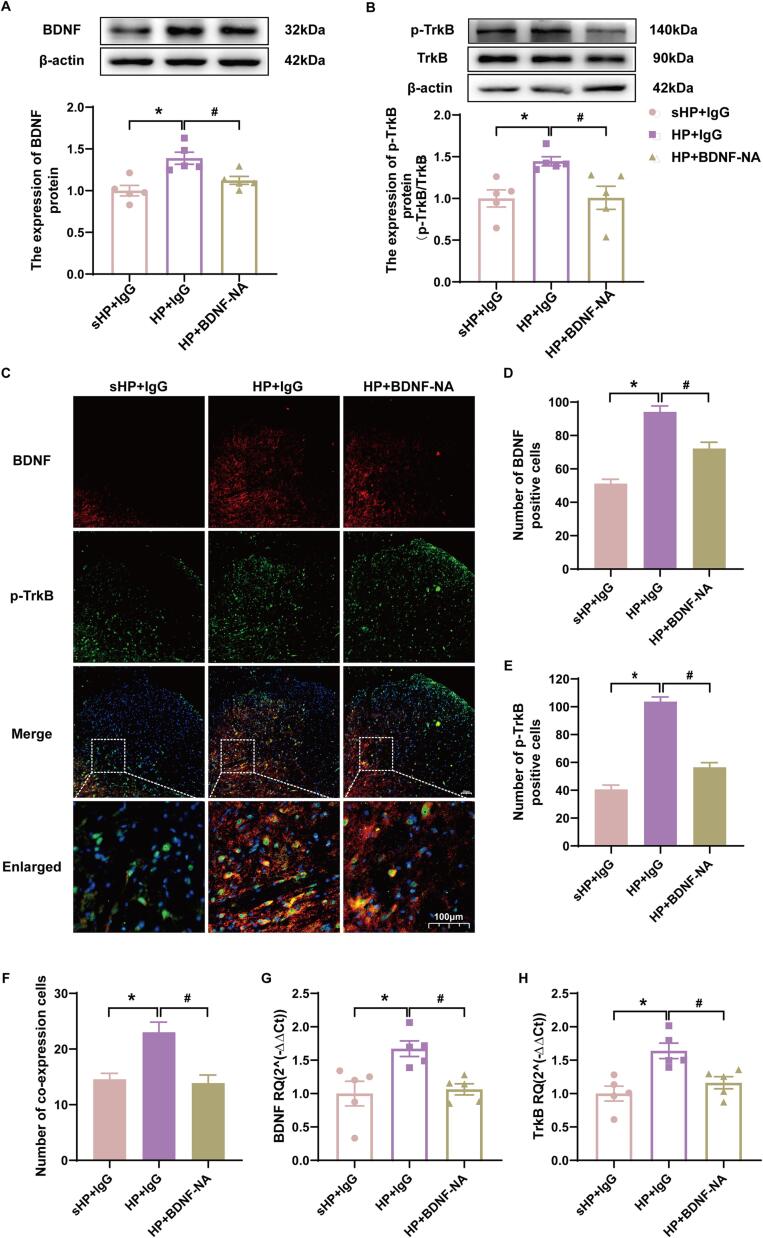
BDNF neutralizing antibody intervention reduced phosphorylation of TrkB. (A-B) The quantification of the Western blots results and a representative Western blot showing BDNF and p-TrkB protein isolated from the ipsilateral SCDH 48 h after 2nd injection. (C) Representative immunofluorescence images of BDNF (red) merged with p-TrkB (green) in ipsilateral L4-6 SCDH of BDNF neutralizing antibody intervention rats. (D-F) The immunofluorescence quantification of BDNF and p-TrkB co-expression cells in ipsilateral L4-6 SCDH. (G-H) Evaluation of BDNF and p-TrkB gene expression in ipsilateral L4-6 SCDH using qPCR in the sHP + IgG, HP + IgG, HP + BDNF neutralizing antibody groups 48 h after 2nd injection. Data are presented as mean ± SEM, n = 5; *p < 0.05, **p < 0.01 vs sHP + IgG group, ^#^*p* < 0.05, ^##^*p* < 0.01 vs HP + IgG group. Two-way ANOVA with Tukey's post-hoc test was applied in. (For interpretation of the references to colour in this figure legend, the reader is referred to the web version of this article.)

Western blotting showed significant decreased in BDNF and p-TrkB protein expression in the HP + BDNF neutralizing antibody group compared to the HP + IgG group (Fig. 5A-B, *p* < 0.05). Immunofluorescence showed that the number of BDNF and p-TrkB co-expression cells in the ipsilateral SCDH of the HP + BDNF-neutralized antibody group was significantly decreased compared with the HP + IgG group (Fig. 5C-F, *p* < 0.05). In addition, the qPCR results also showed that the gene expression of BDNF and TrkB in the ipsilateral SCDH of the spinal cord decreased significantly after the BDNF-neutralizing antibody intervention (Fig. 5G-H, *p* < 0.05). These results suggested that BDNF in the dorsal horn of the spinal cord of HP model rats regulates KCC2 by regulating TrkB phosphorylation.

### 3.6 BDNF recombinant protein reverses the role of EA in preventing pain transition

**Fig. 6:**
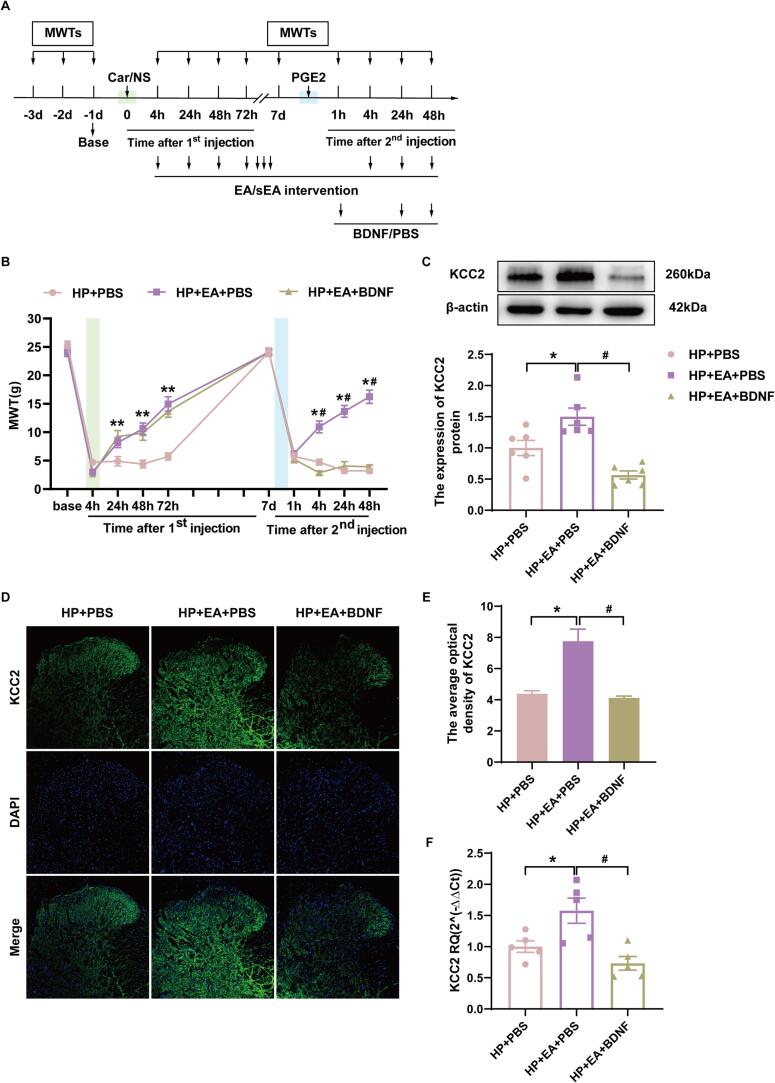
BDNF recombinant protein intervention reversed electroacupuncture relieving the occurrence of hyperalgesic priming. (A)Schedule of establishing hyperalgesic priming model and BDNF recombinant protein intervention. BDNF recombinant protein or PBS was administered through the intrathecal catheter to HP model rats for 3 consecutive days starting on day 7, 1 h before the detection of mechanical withdrawal threshold. (B)The mechanical withdrawal threshold (MWT) of rats that received 3 consecutive days BDNF recombinant protein intervention. (C) The quantification of the Western blots results and a representative Western blot showing KCC2 protein isolated from the ipsilateral SCDH 48 h after 2nd injection. (D)Representative immunofluorescence images of KCC2 (green) merged with Dapi (blue) in ipsilateral L4-6 SCDH of BDNF recombinant protein intervention rats. (E)The immunofluorescence quantification of KCC2 in ipsilateral L4-6 SCDH. (F)Evaluation of KCC2 gene expression in ipsilateral L4-6 SCDH using qPCR in the HP + PBS, HP + EA + PBS, HP + EA + BDNF groups 48 h after 2nd injection. Data are presented as mean ± SEM, n = 5; *p < 0.05, **p < 0.01 vs HP + PBS group, ^#^*p* < 0.05, ^##^*p* < 0.01 vs HP + EA + PBS. Two-way ANOVA with Tukey's post-hoc test was applied in. (For interpretation of the references to colour in this figure legend, the reader is referred to the web version of this article.)

To further investigate the potential mechanism of EA in pain transition, we infused BDNF recombinant protein into the spinal cord intrathecally using intrathecal cannulation. As shown in Fig. 6A, BDNF recombinant protein (0.2 μg/rat/day) or PBS was administered via intrathecal catheter to HP model rats 30 min before EA intervention on 3 consecutive days starting from day 7. Daily intrathecal injections of BDNF recombinant protein significantly and reversed the anti-abnormal pain effects of EA(Fig. 6B, *p* < 0.05). Western blotting showed a significant decrease in KCC2 protein expression in the HP + EA + BDNF group compared to the HP + EA + PBS group (Fig. 6C, *p* < 0.05). Immunofluorescence showed that the mean fluorescence intensity of KCC2 was significantly decreased in the ipsilateral SCDH of rats in the HP + EA + BDNF group compared with the HP + EA + PBS group (Fig. 6D-E, *p* < 0.05). In addition, the qPCR results also showed that the gene expression of KCC2 in the ipsilateral SCDH of the spinal cord decreased significantly after the BDNF recombinant protein intervention (Fig. 6F, *p* < 0.05). BDNF recombinant protein further reversed the effect of EA on regulating KCC2. These results suggest that exogenous administration of BDNF reverses the role of EA in preventing pain transition.

### 3.7 BDNF recombinant protein increased TrkB phosphorylation

**Fig. 7:**
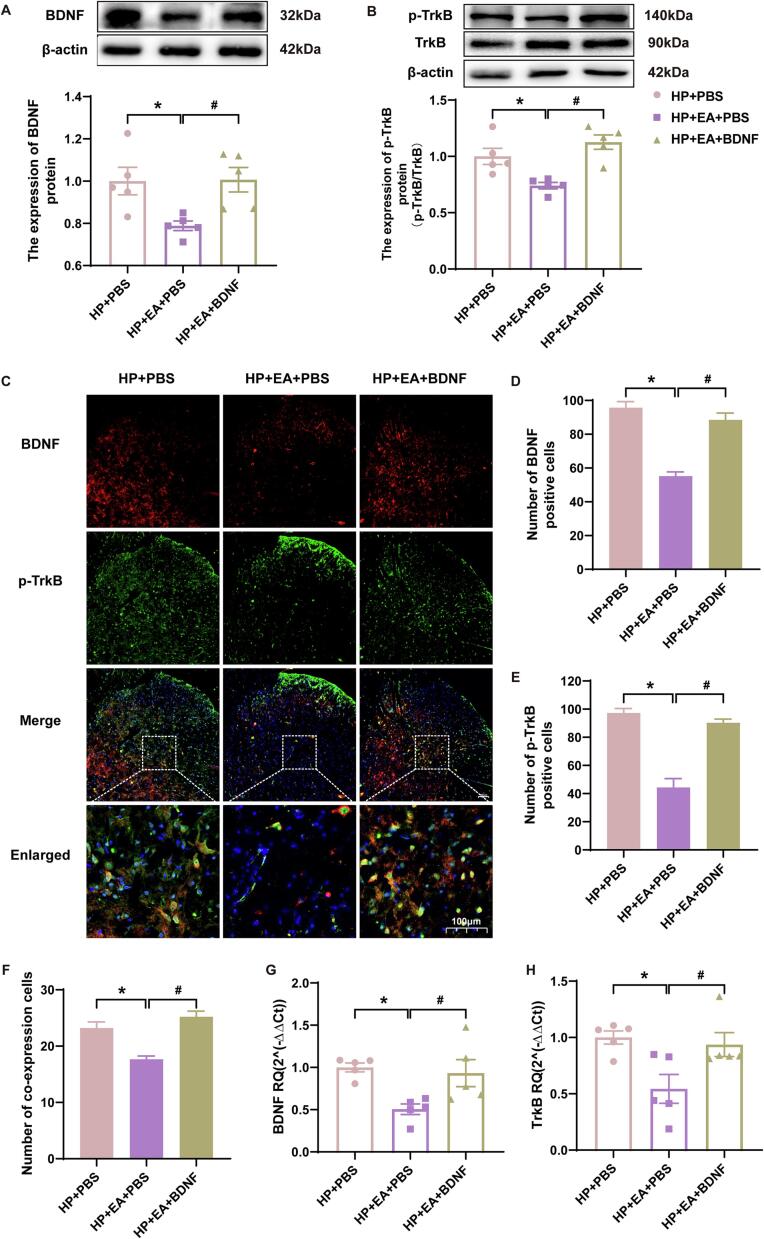
BDNF recombinant protein intervention increased phosphorylation of TrkB. (A-B) The quantification of the Western blots results and a representative Western blot showing BDNF and p-TrkB protein isolated from the ipsilateral SCDH 48 h after 2nd injection. (C) Representative immunofluorescence images of BDNF (red) merged with p-TrkB (green) in ipsilateral L4-6 SCDH of BDNF recombinant protein intervention rats. (D-F) The immunofluorescence quantification of BDNF and p-TrkB co-expression cells in ipsilateral L4-6 SCDH. (G-H) Evaluation of BDNF and p-TrkB gene expression in ipsilateral L4-6 SCDH using qPCR in the HP + PBS, HP + EA + PBS, HP + EA + BDNF groups 48 h after 2nd injection. Data are presented as mean ± SEM, n = 5; *p < 0.05, **p < 0.01 vs HP + PBS group, ^#^*p* < 0.05, ^##^*p* < 0.01 vs HP + EA + PBS group. Two-way ANOVA with Tukey's post-hoc test was applied in. (For interpretation of the references to colour in this figure legend, the reader is referred to the web version of this article.)

Western blotting showed significant increased in BDNF and p-TrkB protein expression in the HP + EA + BDNF group compared to the HP + EA + PBS group (Fig. 7A-B, *p* < 0.05). Immunofluorescence showed that the number of BDNF and p-TrkB co-expression cells in the ipsilateral SCDH of the HP + EA + BDNF group was significantly increased compared with the HP + EA + PBS group (Fig. 7C-F, *p* < 0.05). In addition, the qPCR results also showed that the gene expression of BDNF and TrkB in the ipsilateral SCDH of the spinal cord increased significantly after the BDNF recombinant protein intervention (Fig. 7G-H, *p* < 0.05). These results suggested that BDNF in the dorsal horn of the spinal cord of HP model rats regulates KCC2 by regulating TrkB phosphorylation.

## 4 Discussion

In the present study, we used HP model to investigate the mechanism of transition from acute to chronic pain. We found that nociceptive sensitized rats developed intense and persistent mechanically abnormal pain that could be relieved by daily EA intervention. KCC2 was inhibited in the spinal cord of sensitized rats, accompanied by BDNF overproduction and TrkB hyperphosphorylation. EA intervention significantly activated KCC2 expression in the SCDH of sensitized rats and inhibited BDNF production and TrkB phosphorylation. In addition, BDNF neutralizing antibodies inhibited TrkB phosphorylation, thereby activating KCC2 to suppress pain. Finally, the EA anti-abnormal pain effect and EA-activated KCC2 in the dorsal horn of the spinal cord were blocked by exogenous BDNF. These results suggest that EA ameliorates mechanical abnormal pain in sensitized rats by regulating KCC2 via BDNF-TrkB in the dorsal horn of the spinal cord.

KCC2 has an important role in maintaining neuronal chloride homeostasis and has been found to be associated with many severe neurological, psychiatric and neurodegenerative disorders, including epilepsy, neuropathic pain, schizophrenia, autism spectrum disorders and Parkinson's disease (Ben-Ari, 2017, Silayeva et al., 2015). There is growing evidence that KCC2 is dysregulated when pain occurs and that KCC2 enhancers alleviate spinal cord-mediated nociceptive hyperalgesia (Aby et al., 2022, Gao et al., 2022). In addition, pharmacological blockade of BDNF relieves pain due to chronic pancreatitis and neuropathic pain induced by nerve injury (Hughes et al., 2011, Wang et al., 2021). However, it is unclear whether BDNF and KCC2 are involved in the transition of acute pain into chronic pain. Therefore, we verified the downregulation of KCC2 expression in SCDH of nociceptive sensitized rats by the above mentioned experiments. Our immunofluorescence results determined that KCC2 is mainly expressed in spinal cord neurons and only a few in spinal cord glial cells. This result is consistent with a recent study (Lorenzo et al., 2020). Blocking the pharmacological effects of BDNF by BDNF-neutralizing antibodies significantly improved the abnormal mechanical pain threshold in sensitized rats and normalized KCC2 expression and TrkB phosphorylation. These results suggest that BDNF in the dorsal horn of the rat spinal cord is involved in pain transition through TrkB regulating KCC2.

EA is the most commonly used and effective therapy for clinical analgesia with good efficacy and few side effects (Seo et al., 2017, He et al., 2022). However, the underlying mechanisms of EA against pain transition are still largely unknown. In our study, we chose an EA frequency of 2/100 Hz and set out to explore the mechanisms of antianalgesia in sensitized rats. We found that EA significantly reduced BDNF production and TrkB phosphorylation and promoted expression of KCC2 in the spinal cord of sensitized rats. However, whether EA is involved in pain transition mechanisms through the BDNF-TrkB pathway is still unknown. Therefore, in the present study, we once again conducted pharmacological experiments to confirm that exogenous BDNF reversed the effects of EA on anti-abnormal pain and on regulating KCC2 in sensitized rats. Together, these results confirm a causal relationship between pain responses and expression of KCC2 in the spinal cord of nociceptive sensitized rats. Since we found that KCC2 is expressed primarily in neurons in the SCDH, we then proceeded to examine whether EA can activate KCC2 in these neurons in the SCDH. Both immunoblotting and immunostaining showed that EA significantly activated KCC2 expression in sensitized rat SCDH and inhibited BDNF production and TrkB phosphorylation. Behavioral combined with pharmacological studies further showed that the EA-induced anti-abnormal pain effects could be reversed by activating BDNF. Thus, our results suggest that EA reduces mechanical abnormal pain in nociceptive sensitized rats through BDNF-TrkB regulating KCC2 in spinal dorsal horn neurons.

## Data availability

All the data used to support the findings of this study are included within the article.

## Funding statement

This work was supported by the National Natural Science Foundation of China (No.82174487); Zhejiang Provincial Program for the Cultivation of High-level Innovative Health Talents.

## Declaration of Competing Interest

The authors declare that they have no known competing financial interests or personal relationships that could have appeared to influence the work reported in this paper.
